# Roles of low muscle strength and sarcopenic obesity on incident symptomatic knee osteoarthritis: A longitudinal cohort study

**DOI:** 10.1371/journal.pone.0311423

**Published:** 2024-10-03

**Authors:** Laijun Yan, Haiya Ge, Zhengming Wang, Anping Shen, Qinguang Xu, Ding Jiang, Yuelong Cao

**Affiliations:** Shi’s Center of Orthopedics and Traumatology, Shuguang Hospital Affiliated to Shanghai University of Traditional Chinese Medicine, Shanghai, China; Fondazione Don Carlo Gnocchi, ITALY

## Abstract

**Objectives:**

Sarcopenia is prevalent in middle to old age. We aimed to investigate the association between muscle strength and the incident knee osteoarthritis (OA).

**Methods:**

12,043 participants were collected from the China Health and Retirement Longitudinal Study. The effects of sarcopenic obesity (defined by obesity in combination with possible sarcopenia) on knee OA onset were calculated using Poisson regression models. Mediation analysis was fit to estimate mediating proportion of muscle strength on the association between obesity and incident knee OA.

**Results:**

The study all enrolled 12,043 participants with 2,008 progressed to knee OA. Poisson analyses demonstrated causal association of general obesity (RR:1.23, 95% CI: 1.08 to 1.39) and abdominal obesity (RR:1.23, 95% CI: 1.11 to 1.35) with knee OA onset. For the risk of incident knee OA, participants with the highest level of normalized grip strength had a decreased risk of incident knee OA by 0.33 (RR:0.67, 95% CI: 0.60 to 0.75) times compared to the control group, and chair-rising time was associated with increased risk of incident knee OA by 0.65 (RR:1.65, 95% CI: 1.17 to 2.33) times. Sensitivity analysis identified similar results. Participants with sarcopenic obesity were about 2 times risk of incident knee OA than reference group. Normalized grip strength and chair-rising time mediated the association between obesity and incidence of knee OA.

**Conclusions:**

Sarcopenic obesity is correlated with an increased risk of knee OA. Muscle strength recovery may alleviate the risk of incident knee OA in middle to old age with obesity.

## Introduction

The escalating prevalence of obesity and evolving aging populations are undeniably contributing to a rise in the population at risk for a range of health concerns, including osteoarthritis (OA) [1, 2]. Knee OA is a widespread condition and is responsible for approximately 80% of the global burden of OA [3]. This chronic disease involves the degeneration of the cartilage in the knee joint, leading to symptoms such as pain, stiffness, and reduced mobility [4]. Due to the lack of conservative treatment options for early stage of knee OA, it becomes crucial to clarify the pathophysiology and reduce risk factors.

Sarcopenia is a natural phenomenon of physiological phenomenon defined by the loss of muscle mass and strength that can be accelerated by co-morbid disease states, naturally occurs in ageing [5]. While, obesity increases the risk of developing sarcopenia for chronic inflammation and accumulation of lipids ectopically in skeletal muscle [6]. That generally results to a vicious cycle, as reduced physical activity further contributes to sarcopenic obesity [7]. Several risk factors closely associated with the pathogenesis of KOA have been identified as contributing to the development of sarcopenic obesity. These include low physical activity [8], inflammation [9], and malnutrition [10]. On the other hand, general obesity and abdominal obesity, defined as body mass index (BMI) and waist circumference (WC), are well-known risk factors for both the incidence and progression of knee OA. Cohort studies suggested that a high BMI or WC plays a causative role in the occurrence of knee OA, with a dose-response relationship with the risk for it [11, 12]. It is widely acknowledged that obesity plays a role in the etiology of knee OA by increasing the loading burden on the knee joint, which acts as mechanical stress above the biological capacity of the joints and accelerates the deterioration of cartilage [4]. Given that age-related obesity is also linked to decreased mass and fat infiltration in extremity muscle, from a biomechanics perspective, this condition has a negative impact on the mechanical balance of knee joints [13, 14], suggesting lower muscle strength may mediate the effects of obesity on the onset of knee OA.

Previous studies primarily focused on the association between obesity and OA. However, the absence of obesity does not guarantee a reduced risk of developing knee osteoarthritis, since individuals who are not obese but have low muscle mass may still be at risk for developing knee OA [15]. Recently, a cross-sectional analysis combined with Mendelian randomization study suggested that sarcopenia significantly linked with the risk of OA [16]. Misra, et al. found obesity and sarcopenic obesity but not sarcopenia alone, were associated with an increased risk of knee OA among white adults [17]. This emphasizes the importance of considering both obesity and sarcopenia when assessing the risk of knee OA. Given China’s significant aging population, the association between sarcopenic obesity and the onset of chronic diseases is a matter of great concern [18, 19]. However, research in this topic is still limited. This study investigated the potential effects of muscle strength, sarcopenic obesity on the incidence of knee OA and exploring whether lower muscle strength may mediate the effects of obesity on the onset of it in middle and old age Chinese populations.

## Materials and methods

### Data source

This was a secondary analysis of prospective, longitudinal cohort analysis of baseline and 7-years follow-up data from the China Health and Retirement Longitudinal Study (CHARLS), Available data were accessed on January 2024. CHARLS focusing on adult individuals aged 45 and older, and it was approved by the Biomedical Ethics Review Committee of Peking University (IRB00001052–11015). All participants were required to sign informed consent during the field survey. Details of the CHARLS has been described elsewhere [20]. In the present study, participants were recruited in 2011 wave of CHARLS and re-examined in 2013 or 2015 or 2018. All researchers had no access to information that could identify individual participants.

### Study participants

**Fig 1:**
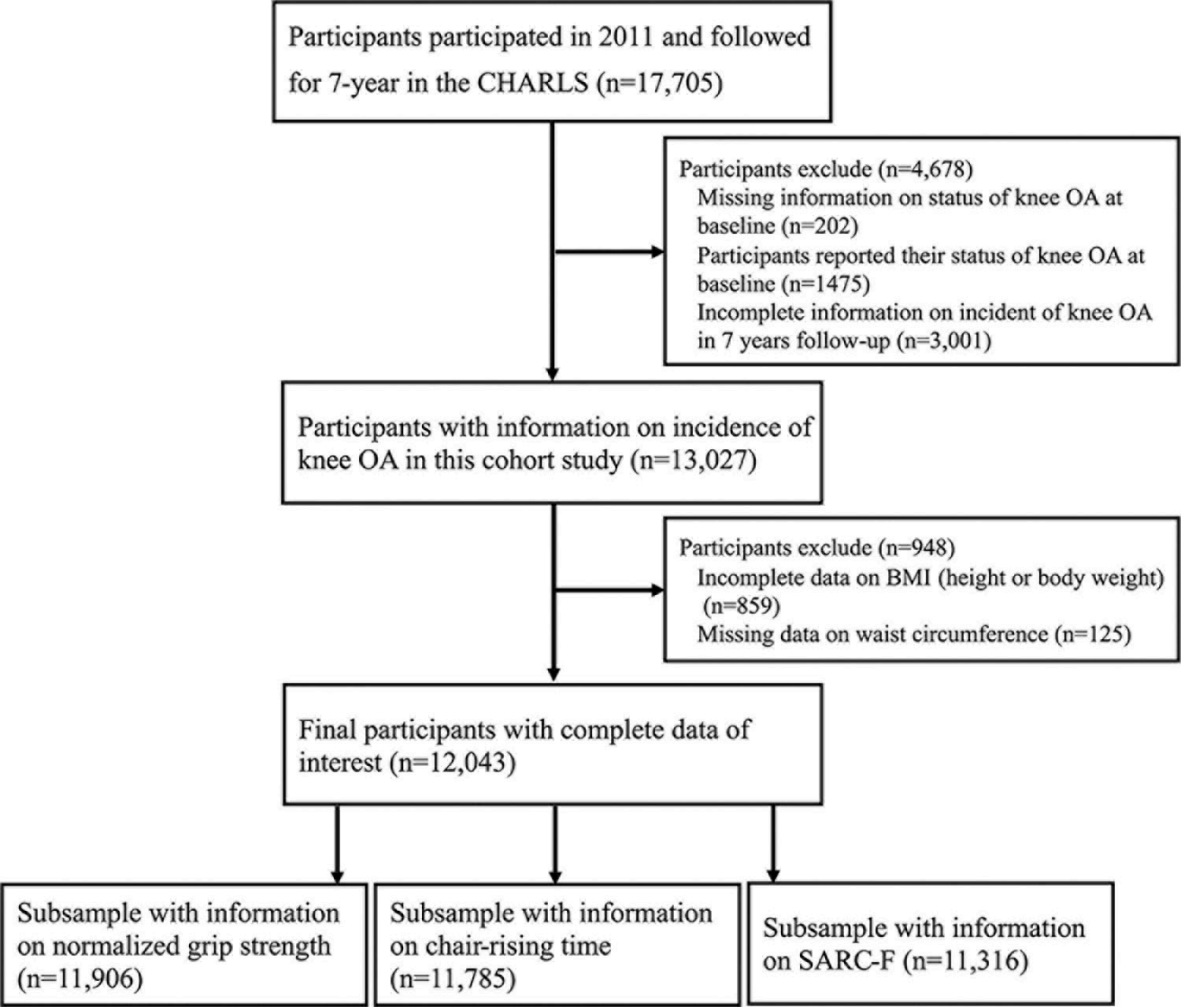
Flow chart of participants selection in CHARLS. Abbreviation: CHARLS, China Health and Retirement Longitudinal Study; OA, osteoarthritis.

Of the 17,005 adults who take part in the 2011 wave of CHARLS, a total of 4,678 were excluded from the study owing to the following reasons: knee OA status was not assessed at baseline, n = 202; confirmed KOA status at baseline, n = 1,475; missing information for KOA diagnose in 7 years follow-up, n = 3,001. We also excluded 859 participants for absent information of BMI, 125 with missing data on WC data, leaving 12,043 participants with eligible for the present analysis (Fig 1).

### Exposure

Data of exposures was obtained from the first wave of CHARLS. Body mass index (BMI) was calculated as weight in kilograms divided by height in meters squared. A general obesity was classified if individuals had a BMI ≥28 kg/m^2^. Waist circumference (WC) was measured using a tape placed around the trunk, measured standing at the iliac crest, crossing at the mid-axillary line. Referring to Zhang, et al. [21], abdominal obesity was classified based on the national guidelines for waist circumference (≥85 cm in females; ≥90 cm in males). For participants reporting no weight change over a three-year period, any missing data for weight, waist circumference and grip strength were imputed from the second wave of CHARLS. Normalized grip strength was calculated as greatest grip strength (kg)/body weight (kg). Chair-rising time was recorded using a stopwatch by guiding participants to stand up and sit down for five repetitions on a chair at their fastest pace. As per the Asian Working Group for Sarcopenia 2019 (AWGS 2019) consensus, a score of simple five-item questionnaire (SARC-F) ≥4, as well as handgrip strength (Male <28 kg, Female <18 kg) or 5-time chair stand test (≥12 s) were used for the screening of “possible sarcopenia” [22]. Sarcopenic obesity was defined as “possible sarcopenia” in combination with general obesity or abdominal obesity.

### Data preprocessing

We conducted outlier detection for continuous variables, including height, weight, waist circumference, and grip strength. Initially, we addressed abnormal values resulting from measurement unit errors. Grip strength values exceeding 170kg, heights below 1m, and weights below 25kg or above 200kg were identified as potential input errors and thus excluded from further analysis. Furthermore, we employed a criterion of 2.5 times the standard deviation to flag outliers based on a normal distribution.

### Outcome assessment

The only outcome was the incident of symptomatic knee OA. Symptomatic knee OA was defined as both physician-diagnosed arthritis and the presence of concurrent pain in either knee joint. Participants were asked about regular body pain on the knee(s), and whether they had physician-diagnosed arthritis. Those who answered affirmatively to both questions were classified as having symptomatic knee OA [23, 24]. The incident of symptomatic knee OA was defined as the participant being free of symptomatic knee OA in CHARLS2011, and diagnosed with symptomatic KOA in CHARLS2013 or CHARLS2015 or CHARLS2018.

### Covariates

Baseline characteristics were collected from the first two survey waves, including age, gender, urban/rural residence area, education level (college or equivalent, middle school or equivalent, primary school, semiliterate, illiteracy), marital status (married, divorced/separated, never married), migrant worker status, physical work status, type of medical insurance, smoking history (100+ cigarettes) and drinking history (1+ times per month). The hypertension diagnosis was established when a systolic blood pressure ≥140 mmHg, or a diastolic blood pressure ≥90 mmHg was detected, or a positive responding to the questionnaire, or the use of antihypertensive agents. The diabetes diagnosis was identified by the questionnaire data, or the detection of a glycosylated hemoglobin ≥6.5%, or the use of antidiabetic drugs. Medical history of dyslipidemia was identified by the questionnaire data, or blood test (triglycerides ≥150 mg/dL, or total cholesterol ≥240 mg/dL, or LDL-cholesterol ≥160 mg/dL, or HDL-cholesterol <40 mg/dL), or the use of anti-dyslipidemic medicine. Regarding comorbidity, the number of physicians diagnosed conditions out of hypertension, diabetes, hypercholesterolemia, cancer, stroke, physical disability, accident injury, and asthma, was categorized as 0, 1~2, and ≥3.

### Statistical analysis

Continuous variables are presented as Mean ± SD for approximately normal distributions, discrete variables are presented as counts (percentages). Independent *t*-tests for continuous variables and chi-square tests for categorical variables were used to compare between-groups difference. We first assessed the longitudinal relation of exposures at baseline to the risk of incident knee OA, using modified Poisson regression with sandwich variance estimator to obtain the relative risk (RR) and 95% confidence intervals (CI) [25]. To examine the dose relationship, exposures were classified into four groups in Poisson regression analyses for trend analysis. Missing data for covariates with extremely low rate (≤1%) were imputed with the mode. Sensitivity analyses were also conducted. First, we excluded participant with abnormal BMI (≥35 or <18.5kg/m^2^). Second, we further excluded participants who reported a history of cancer, physical disability, stoke, and accident injuries. Mediation analysis using bootstrapping (n = 5,000) was utilized to investigate the mediation proportion of biomarkers of muscle strength on the relationships between obesity and onset of knee OA. Considering that the casual effect of obesity on knee OA may primarily rely on muscle strength, we established an ‘obesity–muscle strength–incident knee OA’ model and viewed value of muscle strength as mediator.

Statistical assessments were performed in a two-sided fashion, and a *P* <0.05 was considered statistically significant. All data analysis was conducted using R (version 4.3.1) or SPSS (version 26.0). The “PROCESS” (version 4.1) package developed by Andrew F. Hayes for SPSS was applied for mediation analysis in this study.

## Results

### Baseline characteristics

Of the included 12,043 participants (mean age 58.66 ± 9.85 years), 2,008 developed symptomatic knee OA during the 7-years follow-up (61.1% females). The rate of incident knee OA in middle-old age was 2.2% per year. Baseline characteristics are detailed in Table 1. Compared to those who remained as non-knee OA, those who progressed to symptomatic knee OA had higher prevalence of rural residence, smoking, drinking, hypertension (all *P* <0.05). In the knee OA group, the proportion of participants with lower education level (*P* <0.001), proportion of participants who report their medical insurance as other than “resident insurance” (*P* <0.001), proportion of participants experiencing divorced or separated (*P* <0.001) were significantly higher than that in the non-knee OA group. Moreover, participants who progressed to knee OA had lower normalized grip strength than those who remained as non-knee OA (0.53 ± 0.16 vs. 0.56 ± 0.17, *P* <0.001). In contrast, participants who regressed to knee OA had higher BMI (23.72 ± 0.17 vs. 23.44 ± 3.62, *P* = 0.002), WC (86.08 ± 10.31 vs. 85.47 ± 10.00, *P* <0.001), and chair-rising time (10.87 ± 4.90 vs. 10.32 ± 4.59, *P* <0.001) than those who remained as non-knee OA.

**Table 1:** Basic characteristics of 12,043 participants stratified by incident knee OA.

Variables	Total	Non-knee OA	Knee OA	*P* value
	(n = 12,043)	(n = 10,035)	(n = 2,008)	
**Age (years)** ^**a**^	58.66 ± 9.85	58.60 ± 9.96	58.94 ± 9.27	0.141
**Female (%)** ^**a**^	6230 (51.7)	5003 (49.9)	1227 (61.1)	<0.001
**Rural residence (%)** ^**a**^ ^,^ ^**b**^	9568 (79.4)	7879 (78.5)	1689 (84.1)	<0.001
**Education background (%)** ^**a**^ ^,^ ^**b**^				<0.001
College or equivalent	416 (3.5)	376 (3.7)	40 (2.0)	
Middle school or equivalent	3862 (32.1)	3368 (33.5)	494 (24.6)	
Primary school	2676 (22.2)	2214 (22.1)	462 (23.0)	
Semiliterate	2058 (17.1)	1659 (16.5)	399 (19.9)	
Illiteracy	3031 (25.2)	2418 (24.1)	613 (30.5)	
**Marital status (%)** ^**a**^ ^,^ ^**b**^				<0.001
** Married/cohabiting**	9947 (82.6)	8320 (82.9)	1627 (81.0)	
** Divorced/separated**	854 (7.1)	703 (7.0)	151 (7.5)	
** Never married**	1242 (10.3)	1012 (10.1)	230 (11.5)	
**Medical insurance (%)** ^**b**^				<0.001
Government insurance	1392 (11.6)	1234 (12.3)	158 (7.9)	
Employee insurance	612 (5.1)	523 (5.2)	89 (4.4)	
Resident insurance	9138 (75.9)	7520 (74.9)	1618 (80.6)	
Other	901 (7.5)	758 (7.6)	143 (7.1)	
**Migrant worker (%)** ^**a**^ ^,^ ^**b**^	10736 (89.1)	8949 (89.2)	1787 (89.0)	0.293
**Physical worker (%)** ^**a**^ ^,^ ^**b**^	9023 (74.9)	7514 (74.9)	1509 (75.1)	0.798
**Smoking (%)** ^**a**^ ^,^ ^**b**^	7230 (60.0)	5886 (58.7)	1344 (66.9)	<0.001
**Drinking (%)** ^**a**^ ^,^ ^**b**^	8945 (74.3)	7375 (73.5)	1570 (78.2)	<0.001
**Hypertension (%)** ^**c**^	6957 (57.8)	5744 (57.2)	1213 (60.4)	0.009
**Diabetes (%)** ^**c**^	11182 (92.9)	9311 (92.8)	1871 (93.2)	0.534
**Dyslipidemia (%)** ^**c**^	8395 (69.7)	7016 (69.9)	1379 (68.7)	0.270
**Comorbidities (%)** ^**a**^ ^,^ ^**b**^				0.954
None	7203 (59.8)	5996 (59.8)	1207 (60.1)	
1~2	4493 (37.3)	3749 (37.4)	744 (37.1)	
≥3	347 (2.9)	290 (2.9)	57 (2.8)	
**BMI (kg/m** ^**2**^ **)**	23.48 ± 3.64	23.44 ± 3.62	23.71 ± 3.74	0.002
**WC (cm)**	85.57 ± 10.05	85.47 ± 10.00	86.08 ± 10.31	0.013
**Normalized grip strength** ^**d**^	0.56 ± 0.17	0.56 ± 0.17	0.53 ± 0.16	<0.001
**Chair-rising time (s)** ^**d**^	10.42 ± 4.65	10.32 ± 4.59	10.87 ± 4.90	<0.001

Abbreviation: OA, osteoarthritis; BMI, body mass index; WC, waist circumference

^a^missing information was imputed with mode: 3 for age, 17 for rural resident, 12 for migrant worker, 23 for education background, 13 for marital status, 127 for physical work, 55 for smoke, 60 for drink, 99 for comorbidites.

^b^Status was inferred from related questionnaires

^c^History was obtained by laboratory tests/physical examination and questionnaires (yes or no)

^d^There were 137 and 258 participants with missing information for normalized grip strength and Chair-rising time.

### Association between obesity and incidence of knee OA

As presented in Table 2, participants with higher BMI reported more higher risk of incident knee OA. When we analyzed BMI as categories, general obesity significantly contributed to the development of knee OA in all three models with RRs of 1.23 (95% CI: 1.08 to 1.39), 1.19 (95% CI: 1.05 to 1.35), and 1.16 (95% CI: 1.02 to 1.31), respectively. In the trend analysis, higher BMI groups were significant with incidence to knee OA in the unadjusted model and adjusted models (all *P* <0.05). Similarly, participants with higher WC reported more higher risk of incident knee OA in all models (all *P* <0.05). Participants with abdominal obesity had a higher risk of progression to knee OA, with the risk of incident KOA increasing by 23.0% (RR: 1.23, 95% CI: 1.11 to 1.35), 15% (RR: 1.15, 95% CI: 1.04 to 1.27), and 12.0% (RR: 1.12, 95% CI: 1.01 to 1.24) in all three models (all *P* for trend <0.05). Sensitivity analyses upon the exclusion of participants with an abnormal BMI (<18.5 and ≥35 kg/m^2^) or those with medical history of cancer, physical disability, stoke, and accident injuries were consistent with the primary outcomes (S1 and S2 Tables).

**Table 2:** Poisson regressions for associations between body indices and incident knee OA.

Variables	Model 1^a^	Model 2^b^	Model 3^c^
	RR (95% CIs)	RR (95% CIs)	RR (95% CIs)
**BMI**			
Continuous	1.02 (1.01, 1.03)**	1.02 (1.01, 1.03)**	1.01 (1.00, 1.03)*
Normal	1 (reference)	1 (reference)	1 (reference)
Underweight	1.14 (0.97, 1.35)	1.10 (0.93, 1.29)	1.09 (0.92, 1.29)
Overweight	1.14 (1.04, 1.25)**	1.14 (1.04, 1.25)**	1.12 (1.02, 1.23)*
Obesity	1.23 (1.08, 1.39)**	1.19 (1.05, 1.35)**	1.16 (1.02, 1.31)*
*P* for trend	0.004	0.008	0.030
**Waist circumference**			
Continuous	1.01 (1.00, 1.01) *	1.01 (1.00, 1.01)**	1.01 (1.00, 1.01)*
Lower	1 (reference)	1 (reference)	1 (reference)
Normal	0.95 (0.87, 1.10)	0.94 (0.83, 1.06)	0.93 (0.83, 1.05)
Overweight	1.04 (0.92, 1.17)	0.99 (0.87, 1.11)	0.97 (0.86, 1.10)
Obesity	1.23 (1.11, 1.35)***	1.15 (1.04, 1.27)**	1.12 (1.01, 1.24)**
*P* for trend	<0.001	0.009	0.031

Abbreviation: OA, osteoarthritis; RR, relative risk; CI, confidence interval; BMI, body mass index.

a Models were unadjusted

b Models were adjusted for gender, age, residence area, marital status, education background, medical insurance, migrant work, physical work, smoking and drinking status

c Models were adjusted for gender, age, residence area, marital status, education background, medical insurance, migrant work, physical work, smoking and drinking status, hypertension, diabetes, dyslipidemia, and comorbidities.

*P < 0.05

**P < 0.01

***P < 0.001.

### Association between muscle strength and incidence of knee OA

The correlation between normalized grip strength and incident knee OA is presented in Table 3. In terms of the risk of incident knee OA, participants in the fourth group (≥0.65) exhibited RRs ranging from 0.67 (95% CI: 0.60 to 0.75) to 0.82 (95% CI: 0.72 to 0.94) when setting the low group (<0.45) as reference (all *P* for trend <0.01) in non-adjusted and adjusted models. Consistently, when we analyzed normalized grip strength as continuous, the risk of new-onset knee OA increased with the elevated value from high to low, respectively. Sensitivity analyses were consistent with the primary outcomes (S1 and S2 Tables).

**Table 3:** Poisson regressions for associations of muscle strength with incident knee OA.

Variables	Model 1^a^	Model 2^b^	Model 3^c^
	RR (95% CIs)	RR (95% CIs)	RR (95% CIs)
**Normalized grip strength**			
Continuous	0.40 (0.31, 0.52)***	0.68 (0.50, 0.93) *	0.66 (0.48, 0.90)**
Low (<0.45)	1 (reference)	1 (reference)	1 (reference)
Normal (0.45~0.55)	0.91 (0.82, 1.01)	0.97 (0.87, 1.08)	0.98 (0.87, 1.09)
Middle (0.55~0.65)	0.75 (0.67, 0.84)***	0.86 (0.76, 0.97)*	0.86 (0.76, 0.98)*
High (≥0.65)	0.67 (0.60, 0.75)***	0.83 (0.73, 0.95)**	0.82 (0.72, 0.94)**
*P* for trend	<0.001	0.001	0.001
**Chair-rising time**			
Continuous	1.02 (1.01, 1.03)***	1.02 (1.01, 1.03)***	1.02 (1.01, 1.03)***
Low (<7.80)	1 (reference)	1 (reference)	1 (reference)
Normal (7.80~9.75)	1.16 (0.83, 1.62)	1.02 (0.74, 1.42)	1.01 (0.73, 1.40)
Middle (9.75~12.30)	1.30 (0.93, 1.81)	1.07 (0.77, 1.49)	1.07 (0.77, 1.49)
High (≥12.30)	1.65 (1.17, 2.33)**	1.34 (0.95, 1.89)	1.39 (0.98, 1.95)
*P* for trend	<0.001	<0.001	<0.001

Abbreviation: OA, osteoarthritis; RR, relative risk; CI, confidence interval.

a Models were unadjusted

b Models were adjusted for gender, age, residence area, marital status, education background, medical insurance, migrant work, physical work, smoking and drinking status

c Models were adjusted for gender, age, residence area, marital status, education background, medical insurance, migrant work, physical work, smoking and drinking status, hypertension, diabetes, dyslipidemia, and comorbidities.

*P < 0.05

**P < 0.01

***P < 0.001.

As showed in Table 3, increasing chair-rising time was positively associated with high risk to incident knee OA (RR: 1.02, 95% CI: 1.01 to 1.03), even after adjusting for multiple covariates in model 2 and model 3 (all *P* <0.001). In group analysis, compared to participants with low chair-rising time (<7.80s), those with high chair-rising time (≥12.30s) had great increased RRs of progression to knee OA in the unadjusted model (RR: 1.65, 95% CI: 1.17 to 2.33, Model 1). In sensitive analysis, the results of regression analysis showed similar results as the primary ones (S1 and S2 Tables).

### Association between sarcopenic obesity and incidence of knee OA

Table 4 shows the results of Poisson regression about the association of sarcopenic obesity with incident knee OA. When obesity was identified by BMI, we found that in the unadjusted model, the RR for incident knee OA were 1.45 (95% CI: 1.22 to 1.74) in sarcopenia group and 2.41 (95% CI: 1.78 to 3.26) in sarcopenic obesity group. The trend analysis results were all significant (all *P* <0.001). Additionally, these associations were still significant after adjustment for other confounders in model 2 and model 3 (all *P* <0.001).

**Table 4:** Poisson regressions for associations of sarcopenic obesity with incident knee OA (revised).

Variables	Model 1^a^	Model 2^b^	Model 3^c^
	RR (95% CIs)	RR (95% CIs)	RR (95% CIs)
**Body mass index**			
Normal	1 (reference)	1 (reference)	1 (reference)
Obesity	1.07 (0.94, 1.22)	1.06 (0.93, 1.21)	1.04 (0.91, 1.19)
Sarcopenia	1.45 (1.22, 1.74) ***	1.36 (1.13, 1.63) **	1.36 (1.128, 1.63) **
Sarcopenic obesity	2.41 (1.78, 3.26) ***	2.13 (1.59, 2.87) ***	2.15 (1.60, 2.89) ***
*P* for trend	<0.001	<0.001	<0.001
**Waist circumference**			
Normal	1 (reference)	1 (reference)	1 (reference)
Obesity	1.20 (1.09, 1.32) ***	1.16 (1.05, 1.27) **	1.14 (1.03, 1.25) **
Sarcopenia	1.54 (1.26, 1.89) ***	1.44 (1.17, 1.78) ***	1.46 (1.18, 1.79) ***
Sarcopenic obesity	1.86 (1.46, 2.37) ***	1.68 (1.32, 2.14) ***	1.65 (1.29, 2.11) ***
*P* for trend	<0.001	<0.001	<0.001

Abbreviation: OA, knee Osteoarthritis; RR, relative risk; CI, confidence interval.

a Models were unadjusted

b Models were adjusted for gender, age, residence area, marital status, education background, medical insurance, migrant work, physical work, smoking and drinking status

c Models were adjusted for gender, age, residence area, marital status, education background, medical insurance, migrant work, physical work, smoking and drinking status, hypertension, diabetes, dyslipidemia, and comorbidities.

*P < 0.05

**P < 0.01

***P < 0.001.

When sarcopenic obesity using the WC definitions, we found that in all three models, abdominal obesity and sarcopenia alone, and sarcopenic obesity were significantly related to incident knee OA compared with the normal group. In the unadjusted models, the RR for incident knee OA were 1.20 (95% CI: 1.09 to 1.31) in abdominal obesity group, 1.54 (95% CI: 1.26 to 1.89) in sarcopenia group, and 1.86 (95% CI: 1.46 to 2.37) in the sarcopenic obesity group. In addition, the RR for trend analysis was also significant (all *P* <0.001). Regression analysis adjusted for other confounders in model 2 and model 3 showed that the primary results did not significantly change (all *P* <0.001) (Table 4). Sensitivity analyses found that those results remained consistent after excluding patients with other reasons for low muscle strength (stroke, physical disorders, cancer) (S3 Table).

### Mediation effect of muscle strength on the associations of obesity with knee OA

**Fig 2:**
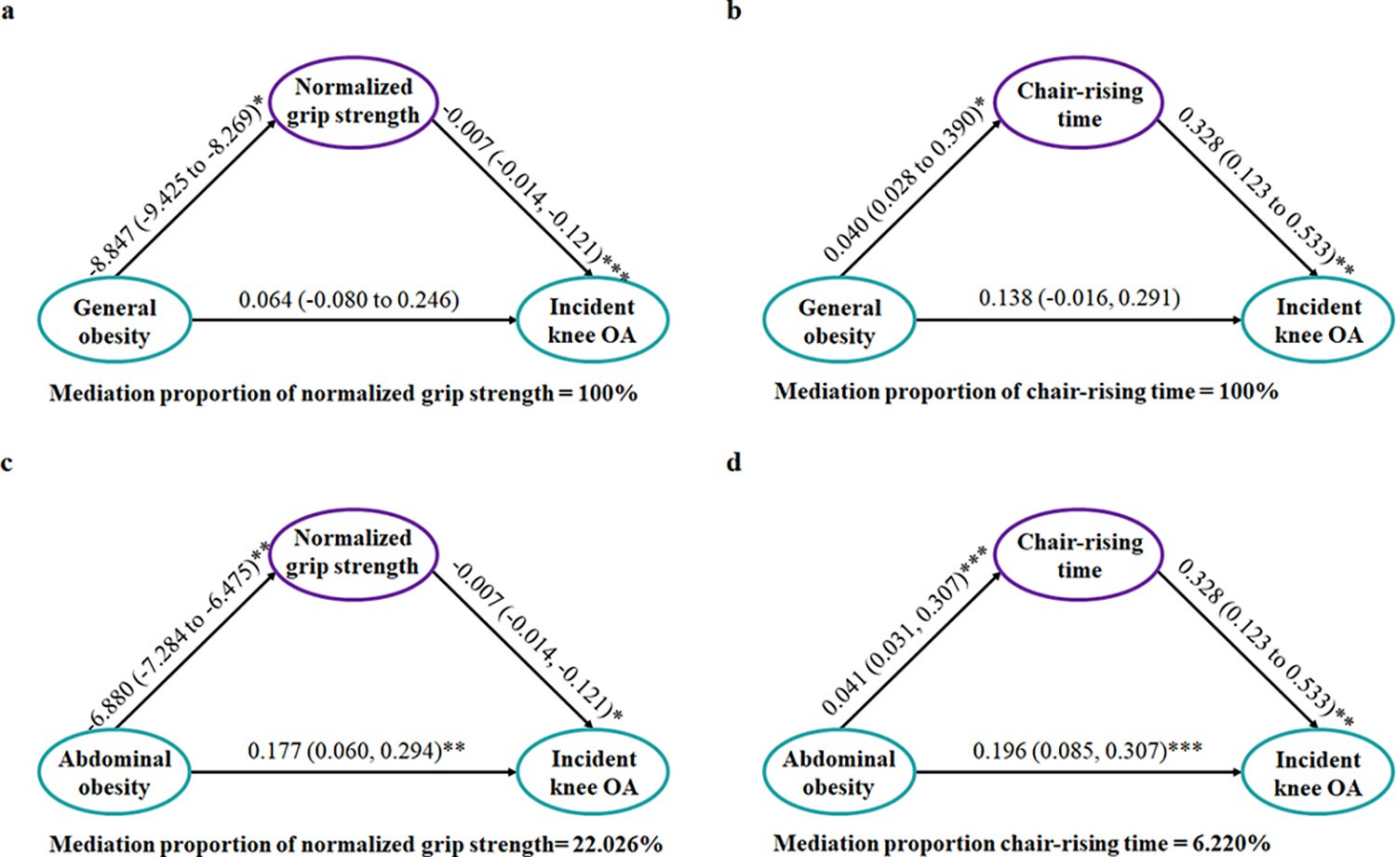
Mediation effect of muscle strength on the associations of obesity with incidence of knee OA. (a, b) all routes except “general obesity to incident knee OA” were significant suggesting normalized grip strength, chair-rising time fully mediated the association of general obesity and incidence of knee OA; (c, d) all routes were significant suggesting normalized grip strength, chair-rising time partially mediated the causation link between abdominal obesity and incidence of knee OA.

Fig 2 and S4 Table present the relationships between general obesity and incident knee OA and between abdominal obesity and incident knee OA, both mediated by normalized grip strength and chair-rising time after adjusted for all potential covariates. After adjusted for all potential covariates, the mediation analysis indicated that the normalized grip strength fully mediated the relationship mediated the correlation between general obesity and incident knee OA (β = 0.064, 95% CI: 0.004 to 0.126). Similarly, the direct effect of the general obesity on the incident knee OA was fully mediated by chair-raising time (β = 0.013, 95% CI: 0.004 to 0.024), indicating a complete mediation effect. Likewise, the mediating effect of muscle strength on the correlation between abdominal obesity and incident knee OA was significant (β = 0.050, 95% CI: 0.003 to 0.096), with a proportion of 22.0%. And also, the chair-rising time exhibited a significant mediating effect between the abdominal obesity and incident knee OA (β = 0.013, 95% CI: 0.004 to 0.024), with a proportion of 6.2%.

## Discussion

The objective of this study was to assess the causative link between sarcopenic obesity and incident knee OA, as well as the mediating role of muscle strength on the associations between obesity and the risk of incident knee OA in middle-aged and elderly adults in mainland China. In the current study, with a 4-year follow-up, the estimated incidence of symptomatic knee OA is 2.1% per year, which is similar to our Study [26]. Our findings convey that low muscle strength and sarcopenic obesity are associated with increased risk of incidence of symptomatic knee OA during the 7-years follow-up. Importantly, our study showed for the first time in middle-aged and older Chinese adults that skeletal muscle strength act as a mediator between obesity and symptomatic knee OA incidence.

Obesity overloads the articular surface, causing pathological damage to the cartilages, and hastening the development of knee OA [27]. Additionally, it has been observed that several proinflammatory cytokines released from adipose tissue stimulate joint degradation, increasing knee OA [28]. Positive associations between BMI and progression of knee OA have been widely suggested in previous cohort studies, whereas longitudinal studies have not been able to demonstrate that baseline WC are associated with knee OA outcomes in Chinese adult. As expected, we found general obesity and abdominal obesity were associated with greater risk of incident knee OA. Our results are in accordance with a meta-analysis [29], which enrolled 12 cohort study and revealed that BMI ≥24 kg/m^2^ was identified as a risk factor (OR: 1.29, 95%CI: 1.02 to 1.63). In contrast to our findings, a recent CHARLS study showed no significant association between symptomatic knee OA and BMI or WC over a 4 years follow-up [24]. Upon reviewing their methodology, we noted the absence of data cleaning descriptions for height, weight, and waist circumference, potentially introducing anomalies in BMI and WC data.

The lower extremity muscle generate force to counter external loads, alleviating stress on the knee joints and providing stability during dynamic activities. Recently, a multicenter cohort study demonstrated that patients with knee OA experience atrophy of thigh muscles and an increase in intramuscular fat over time, which lead to a worsening of the patients’ clinical symptoms [30]. A cross-sectional study reported that appendicular skeletal muscle mass was found to be significantly correlated with handgrip strength in elderly Chinese subjects [31]. The chair-rising time serves as an indicator of muscle power, reflecting the strength of quadriceps and gluteus medius, as well as the lower limb balance capability [32]. There is justification to posit that assessments of muscle quality and function, such as normalized grip strength and 5 chair-rising time, provide a more effective measure of functional ability and physical health in the elderly compared to solely considering the decline in muscle quantity [33].Our findings revealed low muscle quality at baseline to be a risk factor for later symptomatic knee OA, even adjusted for potential covariates. To further validate the impact of sarcopenia on the onset of knee OA, we classified participants into four groups (normal, obesity, sarcopenia, and sarcopenic obesity group) based on the criteria of obesity and “possible sarcopenia” definition. Our findings revealed that participants with sarcopenic obesity had about 2 times increased risks for incidence of knee OA, respectively. These results underscore the significant association between obesity and an elevated susceptibility to knee OA, particularly in the context of concurrent sarcopenia. Our research accentuates the importance of considering these diverse aspects of sarcopenia in clinical assessments and intervention. It is crucial to address that sarcopenia is often associated with poor nutritional status and physical performance [10, 34]. Dietary modification to achieve weight reduction and inflammation control where appropriate, together with specific exercises induced weight loss, leads to decrease in intramuscular adipose tissue and impairment of physical activity [35], which in turn may contribute to the cure for knee OA via strength gain and function restoration [36, 37].

National study in South Korea showed the association between general obesity, abdominal obesity and radiographic knee OA were partially mediated by lower extremity muscle mass with a cross-sectional design [38]. In this cohort study, we demonstrated for the first time that muscle quality and function (normalized grip strength and 5-time chair stand test) play a complete mediating role on the association of obesity with incident knee OA. As Qiu et al. reported that normalized grip strength may outperform chair-rising time in predicting future health status [39], we find the mediation proportion of normalized grip strength to obesity and onset knee OA is higher. Understanding the disparities of pathological mechanism on osteoarthritis between general obesity and abdominal obesity is of great importance. Different from general obesity, abdominal obesity extends beyond just mechanical loading. Central adiposity is metabolically active and releases various bioactive molecules known as adipokines [40]. These adipokines, including leptin and IL-1, have been implicated in inflammatory processes and have a direct impact on joint health [41]. Considering low mediation proportion of muscle strength, our results indicated that the causal link between abdominal obesity and incident knee OA may largely attribute to metabolic disorder and inflammation condition.

There were limitations in the design of this studies. First and most importantly, it is well-known that multifaceted nature of sarcopenia encompasses three dimensions: muscle mass, muscle strength, and physical performance [42]. Based on the guidance of the AWGS 2019, we defined the “possible sarcopenia”, which is recommended in primary health care or community preventive services settings, for the identifying of sarcopenia. While utilizing the SARC-F questionnaire for case finding, along with grip strength measurements and the 5-time chair stand test to assess muscle strength and physical function, holds significance as clinical tools [43, 44], but they cannot fully replace the assessment of muscle mass. Therefore, further research in this area is needed to enhance the diagnosis of sarcopenic obesity. Secondly, our study showed similar results from the analyses between chair-rising time and normalized grip strength, yet it remains to be investigated whether muscle quality and quantity defined by other measures such as fat free mass or muscle mass would yield different conclusion. Thirdly, this study was limited by the diagnostic methods and lacking of imaging-based classification for knee OA. However, a researcher showed 81% consistency between self-reported OA and clinically confirmed OA [45]. Peeters et al. also found that the pooled sensitivity and specificity suggested that the validity of self-reported arthritis is acceptable for use in large-scale studies [46]. Lastly, it is possible that other competing exposures could partially explain the observed association between normalized grip strength and favorable knee health status. We therefore urge caution generalizing our results to the knee OA population at global large, as greater heterogeneity in race or ethnicity [47], weather conditions [48] would likely interact the longitudinal associations.

## Conclusion

Low muscle strength and sarcopenic obesity contribute to incident symptomatic knee OA in middle-aged and older Chinese adults. Our results indicate that improving muscle strength may be a more effective strategy to onset of knee OA in Chinese middle-age to old adults with general and/or abdominal obesity. Interventional studies are warranted to explore whether reducing body fat combined with muscle strength recovery could better prevent knee OA.

## Supporting information

S1 Table Poisson regressions for associations of risk variables with incident knee OA after excluding participants with abnormal BMI (18.0< or >35 kg/m^2^). Abbreviation: OA, osteoarthritis; RR, relative risk; CI, confidence interval; BMI, body mass index. a Models were unadjusted; b Models were adjusted for gender, age, residence area, marital status, education background, medical insurance, migrant work, physical work, smoking and drinking status; c Models were adjusted for gender, age, residence area, marital status, education background, medical insurance, migrant work, physical work, smoking and drinking status, hypertension, diabetes, dyslipidemia, and comorbidities. *P < 0.05 **P < 0.01, ***P < 0.001.
(DOCX)

S2 Table Poisson regressions for associations of risk variables with incident knee OA after excluding participants with cancer, physical disability, stoke, injuries and abnormal BMI (18.0< or >35 kg/m2). Abbreviation: OA, osteoarthritis; RR, relative risk; CI, confidence interval; BMI, body mass index. a Models were unadjusted; b Models were adjusted for gender, age, residence area, marital status, education background, medical insurance, migrant work, physical work, smoking and drinking status; c Models were adjusted for gender, age, residence area, marital status, education background, medical insurance, migrant work, physical work, smoking and drinking status, hypertension, diabetes, dyslipidemia, and comorbidities. *P < 0.05 **P < 0.01, ***P < 0.001.
(DOCX)

S3 Table Poisson regressions for associations of sarcopenic obesity with incident knee OA after excluding participants with cancer, physical disability, stroke. Abbreviation: OA, osteoarthritis; RR, relative risk; CI, confidence interval. a Models were unadjusted; b Models were adjusted for gender, age, residence area, marital status, education background, medical insurance, migrant work, physical work, smoking and drinking status; c Models were adjusted for gender, age, residence area, marital status, education background, medical insurance, migrant work, physical work, smoking and drinking status, hypertension, diabetes, dyslipidemia, and comorbidities. *P < 0.05 **P < 0.01, ***P < 0.001.
(DOCX)

S4 Table Mediation of muscle strength on the association between obesity and knee OA. All models were adjusted for gender, age, residence area, marital status, education background, medical insurance, migrant work, physical work, smoking and drinking status, hypertension, diabetes, dyslipidemia, and comorbidities; Proportion of mediation = (a*b/(c’+a*b)) *100%; *P < 0.05 **P < 0.01, ***P < 0.001.
(DOCX)
