# Supplementary material for: Roles of low muscle strength and sarcopenic obesity on incident symptomatic knee osteoarthritis: A longitudinal cohort study
Source: PLoS One. 2024 Oct 3;19(10):e0311423. doi: 10.1371/journal.pone.0311423 (PMC11449331; doi:10.1371/journal.pone.0311423)
Supplement: S1 Table — Abbreviation: OA, osteoarthritis; RR, relative risk; CI, confidence interval; BMI, body mass index. a Models were unadjusted; b Models were adjusted for gender, age, residence area, marital status, education background, medical insurance, migrant work, physical work, smoking and drinking status; c Models were adjusted for gender, age, residence area, marital status, education background, medical insurance, migrant work, physical work, smoking and drinking status, hypertension, diabetes, dyslipidemia, and comorbidities. *P < 0.05 **P < 0.01, ***P < 0.001. (DOCX) [file pone.0311423.s001.docx]

**S1 Table. Poisson regressions for associations of risk variables with incident knee OA after excluding participants with abnormal BMI (18.0< or >35 kg/m^2^)**

| **Variables** | **Model 1^a^** | **Model 2^b^** | **Model 3^c^** |
| --- | --- | --- | --- |
|  | **RR (95% CIs)** | **RR (95% CIs)** | **RR (95% CIs)** |
| **BMI** |  |  |  |
| Continuous | 1.03 (1.01, 1.04)*** | 1.02 (1.01, 1.04)*** | 1.02 (1.00, 1.03)*** |
| Normal | 1 (reference) | 1 (reference) | 1 (reference) |
| Overweight | 1.14 (1.04, 1.25)** | 1.14 (1.04, 1.25)** | 1.12 (1.02, 1.23)* |
| Obesity | 1.23 (1.08, 1.39)** | 1.19 (1.05, 1.35)** | 1.17 (1.03, 1.33)* |
| *P* for trend | 1.12 (1.05, 1.18)*** | 1.10 (1.04, 1.17)*** | 1.09 (1.03, 1.16)** |
| **Waist circumference** |  |  |  |
| Continuous | 1.01 (1.00, 1.01)* | 1.01 (1.00, 1.01)** | 1.01 (1.00, 1.01)* |
| Lower | 1 (reference) | 1 (reference) | 1 (reference) |
| Normal | 0.99 (0.86, 1.12) | 0.94 (0.86, 1.06) | 0.99 (0.82, 1.06) |
| Overweight | 1.05 (0.93, 1.19) | 0.99 (0.87, 1.12) | 0.97 (0.86, 1.11) |
| Obesity | 1.25 (1.13, 1.38)*** | 1.14 (1.03, 1.27)* | 1.12 (1.01, 1.24)* |
| *P* for trend | 1.08 (1.04, 1.11)*** | 1.05 (1.01, 1.08)* | 1.04 (1.00, 1.07)* |
| **Normalized grip strength** |  |  |  |
| Continuous | 0.35 (0.27, 0.46)*** | 0.61 (0.44, 0.85)** | 0.612 (0.44, 0.86)** |
| Low (<0.45) | 1 (reference) | 1 (reference) | 1 (reference) |
| Normal (0.45~0.55) | 0.91 (0.82, 1.01) | 0.96(0.86, 1.07) | 0.961 (0.86, 1.08) |
| Middle (0.55~0.65) | 0.75 (0.67, 0.84)*** | 0.86 (0.76, 0.97)* | 0.86 (0.76, 0.97)* |
| High (≥0.65) | 0.64 (0.57, 0.72)*** | 0.79 (0.69, 0.92)** | 0.79 (0.69, 0.92)** |
| *P* for trend | 0.86 (0.83, 0.89)*** | 0.92 (0.88, 0.97)*** | 0.92 (0.88, 0.97)*** |
| **Chair-rising time** |  |  |  |
| Continuous | 1.02 (1.014, 1.03)*** | 1.02 (1.01, 1.02)*** | 1.02 (1.01, 1.03)*** |
| Low (<7.80) | 1 (reference) | 1 (reference) | 1 (reference) |
| Normal (7.80~9.75) | 1.09 (0.78, 1.52) | 0.95 (0.68, 1.31) | 0.96 (0.69, 1.33) |
| Middle (9.75~12.30) | 1.22 (0.87, 1.70) | 0.98 (0.71, 1.37) | 0.99 (0.71, 1.38) |
| High (≥12.30) | 1.58 (1.12, 2.22)** | 1.25 (0.88, 1.76) | 1.30 (0.92, 1.84) |
| *P* for trend | <0.001 | <0.001 | <0.001 |

Abbreviation: OA, osteoarthritis; RR, relative risk; CI, confidence interval; BMI, body mass index.

^a^Models were unadjusted;

^b^Models were adjusted for gender, age, residence area, marital status, education background, medical insurance, migrant work, physical work, smoking and drinking status;

^c^Models were adjusted for gender, age, residence area, marital status, education background, medical insurance, migrant work, physical work, smoking and drinking status, hypertension, diabetes, dyslipidemia, and comorbidities.

**P* < 0.05 ***P* < 0.01, ****P* < 0.001.
