# Supplementary material for: Roles of low muscle strength and sarcopenic obesity on incident symptomatic knee osteoarthritis: A longitudinal cohort study
Source: PLoS One. 2024 Oct 3;19(10):e0311423. doi: 10.1371/journal.pone.0311423 (PMC11449331; doi:10.1371/journal.pone.0311423)
Supplement: S2 Table — Abbreviation: OA, osteoarthritis; RR, relative risk; CI, confidence interval; BMI, body mass index. a Models were unadjusted; b Models were adjusted for gender, age, residence area, marital status, education background, medical insurance, migrant work, physical work, smoking and drinking status; c Models were adjusted for gender, age, residence area, marital status, education background, medical insurance, migrant work, physical work, smoking and drinking status, hypertension, diabetes, dyslipidemia, and comorbidities. *P < 0.05 **P < 0.01, ***P < 0.001. (DOCX) [file pone.0311423.s002.docx]

**S2 Table. Poisson regressions for associations of risk variables with incident knee OA after excluding participants with cancer, physical disability, stoke, injuries and abnormal BMI (18.0< or >35 kg/m^2^)**

| **Variables** | **Model 1^a^** | **Model 2^b^** | **Model 3^c^** |
| --- | --- | --- | --- |
|  | **RR (95% CIs)** | **RR (95% CIs)** | **RR (95% CIs)** |
| **BMI** |  |  |  |
| Continuous | 1.02 (1.01, 1.04)*** | 1.02 (1.01, 1.04)** | 1.02 (1.00, 1.03)** |
| Normal | 1 (reference) | 1 (reference) | 1 (reference) |
| Overweight | 1.13 (1.02, 1.25)* | 1.13 (1.02, 1.24)* | 1.11 (1.00, 1.22)* |
| Obesity | 1.18 (1.03, 1.36)* | 1.14 (0.99, 1.31) | 1.11 (0.96, 1.28) |
| *P* for trend | 1.10 (1.03, 1.17)** | 1.08 (1.02, 1.15)* | 1.07 (1.00, 1.14)* |
| **Waist circumference** |  |  |  |
| Continuous | 1.01 (1.00, 1.01)* | 1.006 (1.001, 1.01)* | 1.01 (1.00, 1.01) |
| Lower | 1 (reference) | 1 (reference) | 1 (reference) |
| Normal | 1.00 (0.88, 1.15) | 0.951 (0.83, 1.09) | 0.94 (0.82, 1.08) |
| Overweight | 1.03 (0.90, 1.18) | 0.967 (0.84, 1.11) | 0.95 (0.83, 1.10) |
| Obesity | 1.23 (1.10, 1.37)*** | 1.132 (1.01, 1.27)* | 1.10 (0.98, 1.24) |
| *P* for trend | 1.07 (1.03, 1.11)*** | 1.041 (1.00, 1.08)* | 1.03 (0.99, 1.07) |
| **Normalized grip strength** |  |  |  |
| Continuous | 0.38 (0.28, 0.51)*** | 0.649 (0.45, 0.94)* | 0.66 (0.46, 0.95)* |
| Low (<0.45) | 1 (reference) | 1 (reference) | 1 (reference) |
| Normal (0.45~0.55) | 0.95 (0.84, 1.07) | 1.00 (0.88, 1.15) | 1.00 (0.89, 1.13) |
| Middle (0.55~0.65) | 0.77 (0.68, 0.87)*** | 0.88 (0.77, 1.01) | 0.88 (0.77, 1.01) |
| High (≥0.65) | 0.66 (0.58, 0.76)*** | 0.81 (0.69, 0.95)** | 0.82 (0.70, 0.96)* |
| *P* for trend | 0.87 (0.83, 0.90)*** | 0.93 (0.89, 0.98)** | 0.93 (0.89, 0.98)** |
| **Chair-rising time** |  |  |  |
| Continuous | 1.024 (1.02, 1.03)*** | 1.02 (1.01, 1.03)*** | 1.020 (1.011, 1.03)*** |
| Low (<7.80) | 1 (reference) | 1 (reference) | 1 (reference) |
| Normal (7.80~9.75) | 1.12 (0.78, 1.61) | 0.98 (0.69, 1.41) | 0.99 (0.69, 1.42) |
| Middle (9.75~12.30) | 1.25 (0.87, 1.80) | 1.03 (0.72, 1.49) | 1.04 (0.72, 1.50) |
| High (≥12.30) | 1.68 (1.15, 2.44)** | 1.35 (0.93, 1.95) | 1.41 (0.97, 2.06) |
| *P* for trend | 1.19 (1.12, 1.27)*** | 1.13 (1.06, 1.21)*** | 1.14 (1.07, 1.22)*** |

Abbreviation: OA, osteoarthritis; RR, relative risk; CI, confidence interval; BMI, body mass index.

^a^Models were unadjusted;

^b^Models were adjusted for gender, age, residence area, marital status, education background, medical insurance, migrant work, physical work, smoking and drinking status;

^c^Models were adjusted for gender, age, residence area, marital status, education background, medical insurance, migrant work, physical work, smoking and drinking status, hypertension, diabetes, dyslipidemia, and comorbidities.

**P* < 0.05 ***P* < 0.01, ****P* < 0.001.
