# Supplementary material for: Roles of low muscle strength and sarcopenic obesity on incident symptomatic knee osteoarthritis: A longitudinal cohort study
Source: PLoS One. 2024 Oct 3;19(10):e0311423. doi: 10.1371/journal.pone.0311423 (PMC11449331; doi:10.1371/journal.pone.0311423)
Supplement: S3 Table — Abbreviation: OA, osteoarthritis; RR, relative risk; CI, confidence interval. a Models were unadjusted; b Models were adjusted for gender, age, residence area, marital status, education background, medical insurance, migrant work, physical work, smoking and drinking status; c Models were adjusted for gender, age, residence area, marital status, education background, medical insurance, migrant work, physical work, smoking and drinking status, hypertension, diabetes, dyslipidemia, and comorbidities. *P < 0.05 **P < 0.01, ***P < 0.001. (DOCX) [file pone.0311423.s003.docx]

**S3 Table. Poisson regressions for associations of sarcopenic obesity with incident knee OA after excluding participants with cancer, physical disability, stroke**

| **Variables** | **Model 1^a^** | **Model 2^b^** | **Model 3^c^** |
| --- | --- | --- | --- |
|  | **RR (95% CIs)** | **RR (95% CIs)** | **RR (95% CIs)** |
| **BMI** |  |  |  |
| Normal | 1 (reference) | 1 (reference) | 1 (reference) |
| Obesity | 1.08 (0.94, 1.24) *** | 1.07 (0.93, 1.23) | 1.04 (0.91, 1.20) |
| Sarcopenia | 1.55 (1.34, 1.80) *** | 1.43 (1.23, 1.66) *** | 1.43 (1.23. 1.66) *** |
| Sarcopenic obesity | 2.21 (1.69, 2.89) *** | 1.92 (1.48, 2.50) *** | 1.92 (1.48, 2.50) *** |
| *P* for trend | <0.001 | <0.001 | <0.001 |
| **Waist circumference** |  |  |  |
| Normal | 1 (reference) | 1 (reference) | 1 (reference) |
| Obesity | 1.19 (1.08, 1.31) *** | 1.15 (1.04, 1.27) ** | 1.13 (1.02, 1.24) * |
| Sarcopenia | 1.63 (1.38, 1.93) *** | 1.50 (1.27, 1.78) *** | 1.51 (1.28, 1.79) *** |
| Sarcopenic obesity | 1.86 (1.52, 2.28) *** | 1.65 (1.35, 2.03) *** | 1.62 (1.32, 1.99) *** |
| *P* for trend | <0.001 | <0.001 | <0.001 |

Abbreviation: OA, Osteoarthritis; RR, relative risk; CI, confidence interval.

a Models were unadjusted;

b Models were adjusted for gender, age, residence area, marital status, education background, medical insurance, migrant work, physical work, smoking and drinking status;

c Models were adjusted for gender, age, residence area, marital status, education background, medical insurance, migrant work, physical work, smoking and drinking status, hypertension, diabetes, dyslipidemia, and comorbidities.

**P* < 0.05 ***P* < 0.01, ****P* < 0.001.
