# Supplementary material for: Roles of low muscle strength and sarcopenic obesity on incident symptomatic knee osteoarthritis: A longitudinal cohort study
Source: PLoS One. 2024 Oct 3;19(10):e0311423. doi: 10.1371/journal.pone.0311423 (PMC11449331; doi:10.1371/journal.pone.0311423)
Supplement: S4 Table — All models were adjusted for gender, age, residence area, marital status, education background, medical insurance, migrant work, physical work, smoking and drinking status, hypertension, diabetes, dyslipidemia, and comorbidities; Proportion of mediation = (a*b/(c’+a*b)) *100%; *P < 0.05 **P < 0.01, ***P < 0.001. (DOCX) [file pone.0311423.s004.docx]

**S4 Table. Mediation of muscle strength on the association between obesity and knee OA**

| **Path** | **Mediator:** **normalized grip strength** | | | **Mediators: chair-rising time** | | |
| --- | --- | --- | --- | --- | --- | --- |
|  | **β** | **95% CI** | ***P* value** | **β** | **95% CI** | ***P* value** |
| **General obesity** |  |  |  |  |  |  |
| a (obesity to mediator) | -8.847 | -9.425, -8.269 | 0.0000 | 0.040 | 0.028, 0.390 | 0.0146 |
| b (mediator to KOA) | -0.007 | -0.014, -0.121 | 0.0000 | 0.328 | 0.123, 0.533 | 0.0017 |
| c’ (obesity to KOA) | 0.083 | -0.080, -0.246 | 0.3175 | 0.138 | -0.016, 0.291 | 0.2909 |
| Indirect effect (a*b) | 0.064 | 0.004, 0.126 | - | 0.013 | 0.004, 0.024 | - |
| Proportion (%) | - | | | - | | |
| **Abdominal obesity** |  |  |  |  |  |  |
| a (obesity to mediator) | -6.880 | -7.284, -6.475 | 0.0031 | 0.041 | 0.031, 0.307 | 0.0005 |
| b (mediator to KOA) | -0.007 | -0.014, -0.121 | 0.0268 | 0.328 | 0.123, 0.533 | 0.0017 |
| c’ (obesity to KOA) | 0.177 | 0.060, 0.294 | 0.0031 | 0.196 | 0.085, 0.307 | <0.001 |
| Indirect effect (a*b) | 0.050 | 0.003, 0.096 | - | 0.013 | 0.004, 0.024 | - |
| Proportion (%) | 22.026 | | | 6.220 | | |

Abbreviation: OA, Osteoarthritis; CI, confidence interval.

All models were adjusted for gender, age, residence area, marital status, education background, medical insurance, migrant work, physical work, smoking and drinking status, hypertension, diabetes, dyslipidemia, and comorbidities;

Proportion of mediation= (a*b/(c’+a*b)) *100%;

**P* < 0.05 ***P* < 0.01, ****P* < 0.001.
